# Supplementary material for: The Small RNA Universe of Capitella teleta
Source: Front Mol Biosci. 2022 Feb 25;9:802814. doi: 10.3389/fmolb.2022.802814 (PMC8915122; doi:10.3389/fmolb.2022.802814)
Supplement: Supplementary file 1 [file DataSheet1.ZIP › Supplement/known/CAPTEscaffold_24_2926.pdf]

Provisional ID	:	CAPTEscaffold_24_2926
Score total	:	6322.3
Score for star read(s)	:	3.9
Score for read counts	:	6313.3
Score for mfe	:	0.5
Score for randfold	:	1.6
Score for cons. seed	:	3
Total read count	:	12395
Mature read count	:	11972
Loop read count	:	0
Star read count	:	423

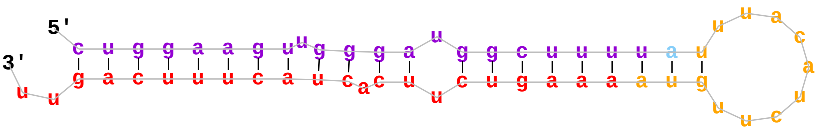

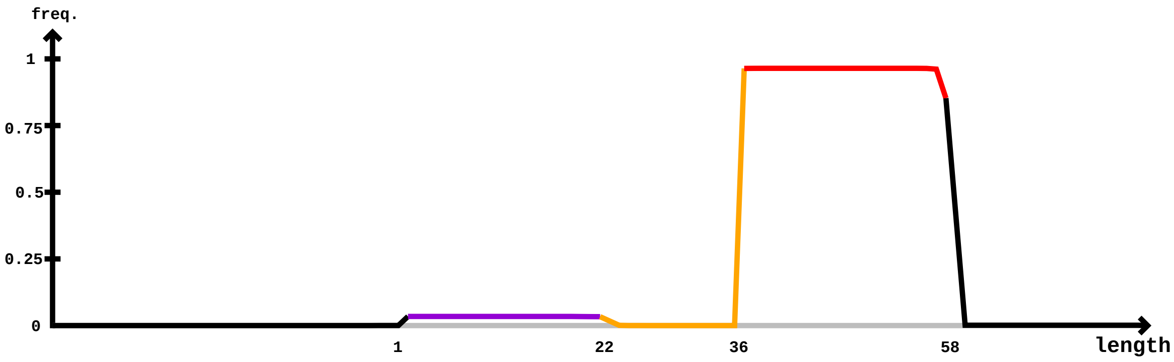

Star Mature

```
5'- ugcauguaaccaauacuaaucuucgcauuucuuaccuggaaguugggauggcuuuuauuuacaucuuguaaaagucuucacuacuuucaguucggagaugugucuuaauacu   -3'   obs
    ugcauguaaccaauacuaaucuucgcauuucuuaccuggaaguugggauggcuuuuauuuacaucuuguaaaagucuucacuacuuucaguucggagaugugucuuaauacu         exp
    ugcauguaaccaauacuaaucuucgcauuucuuaccuggaaguugggauggcuuuuauuuacaucuuguaaaagucuucacuacuuucaguucggagaugugucuuaauacu         known
    .......................(((((((((...((((((((.((((.(((((((((.........))))))))).)).))))))))))...)))))))))..........    reads   mm   sample

    ................................uaccuggaaguugggauggcu...........................................................    1       0    seq
    ................................uaccuggaaguugggauggcuu..........................................................    1       0    seq
    ..................................ccuggaaguugggauggcuuuu........................................................    1       0    seq
    ...................................cuggaaguugggauggcu...........................................................    4       0    seq
    ...................................cuggaaguugggauggcuu..........................................................    3       0    seq
    ...................................cuggaaguugggauggcuuu.........................................................    3       0    seq
    ...................................cuggaaguugggauggcuuuu........................................................    239     0    seq
    ...................................cuggGaguugggauggcuuuu........................................................    2       1    seq
    ...................................cugAaaguugggauggcuuuu........................................................    1       1    seq
    ...................................cuAgaaguugggauggcuuuu........................................................    1       1    seq
    ...................................Uuggaaguugggauggcuuuu........................................................    1       1    seq
    ...................................cuggaaguugggauggcuGuu.........................................................    1       1    seq
    ...................................cuggaaguugggauggcuuuA........................................................    1       1    seq
    ...................................cuggaaguugggauAgcuuuu........................................................    1       1    seq
    ...................................cuggaaguugggauggcCuuu........................................................    1       1    seq
    ...................................cuggaaguugAgauggcuuuua.......................................................    1       1    seq
    ...................................cuAgaaguugggauggcuuuua.......................................................    1       1    seq
    ...................................cuggaaguugggauggcuuuuU.......................................................    1       1    seq
    ...................................cuggaaguuAggauggcuuuua.......................................................    1       1    seq
    ...................................cuggaaguugggauggcuuuuC.......................................................    2       1    seq
    ...................................cuggaaguugggauggcuuuua.......................................................    148     0    seq
    ...................................cuggaaguugggauggcuuuuaA......................................................    4       1    seq
    ...................................cuggaaguugggauggcuuuuau......................................................    5       0    seq
    ...................................cuggaaguugggauggcuuuuauuu....................................................    1       0    seq
    ......................................................................aaagucuucacuacuuuca.......................    4       0    seq
    ......................................................................aaagucuucacuacuuucag......................    34      0    seq
    ......................................................................aaagucuAcacuacuuucagu.....................    1       1    seq
    ......................................................................aaagucuucacuacuuucagu.....................    1334    0    seq
    ......................................................................aaagucuuUacuacuuucagu.....................    4       1    seq
    ......................................................................aaagucuucacAacuuucagu.....................    1       1    seq
    ......................................................................aaagucuucacuacuuuUagu.....................    1       1    seq
```

```
                                   Star                             Mature
ugcauguaaccaauacuaaucuucgcauuucuuaccuggaaguugggauggcuuuuauuuacaucuuguaaaagucuucacuacuuucaguucggagaugugucuuaauacu
......................................................................aaagucuuAacuacuuucagu.....................   1      1   seq
......................................................................aaagucuucacuacuuAcagu.....................   1      1   seq
......................................................................aaagucuucacuacuuucagG.....................   2      1   seq
......................................................................aaagucuucacuacuGucagu.....................   1      1   seq
......................................................................aaagucuucacuacuuuAagu.....................   1      1   seq
......................................................................aaagucAucacuacuuucagu.....................   1      1   seq
......................................................................Uaagucuucacuacuuucagu.....................   1      1   seq
......................................................................Naagucuucacuacuuucagu.....................   1      1   seq
......................................................................aaagucuucacuacGuucagu.....................   1      1   seq
......................................................................aaaguAuucacuacuuucagu.....................   1      1   seq
......................................................................aaagucuucacAacuuucaguu....................   6      1   seq
......................................................................aaagucuucacuacuuucagAu....................   5      1   seq
......................................................................aaagucuucacuacuuuGaguu....................   1      1   seq
......................................................................aaaCucuucacuacuuucaguu....................   1      1   seq
......................................................................aaaguAuucacuacuuucaguu....................   1      1   seq
......................................................................Gaagucuucacuacuuucaguu....................   10     1   seq
......................................................................aaagucuucacuGcuuucaguu....................   11     1   seq
......................................................................aaagucuucacuacuuucCguu....................   1      1   seq
......................................................................aaagucuucacuacuuuAaguu....................   5      1   seq
......................................................................aaagucuucacuacAuucaguu....................   2      1   seq
......................................................................aaagucuuUacuacuuucaguu....................   15     1   seq
......................................................................aaagucuucacuacuuuUaguu....................   8      1   seq
......................................................................aaagucuAcacuacuuucaguu....................   2      1   seq
......................................................................aaagucuucacuacuuucaguu....................   10181  0   seq
......................................................................aaagucAucacuacuuucaguu....................   5      1   seq
......................................................................aaagucuucacuacuuAcaguu....................   2      1   seq
......................................................................aaaguUuucacuacuuucaguu....................   2      1   seq
......................................................................aaagucuCcacuacuuucaguu....................   2      1   seq
......................................................................aaagucuucacuacuuucGguu....................   3      1   seq
......................................................................aaagucCucacuacuuucaguu....................   1      1   seq
......................................................................aaagucuucGcuacuuucaguu....................   3      1   seq
......................................................................aaagucGucacuacuuucaguu....................   3      1   seq
......................................................................aaagucuucacuaAuuucaguu....................   2      1   seq
......................................................................aaagucuucacuacuAucaguu....................   7      1   seq
......................................................................aaagucuucacuacuuCcaguu....................   2      1   seq
......................................................................Uaagucuucacuacuuucaguu....................   1      1   seq
......................................................................aaagucuucaAuacuuucaguu....................   1      1   seq
......................................................................aaagucuucacuacuuucagCu....................   1      1   seq
......................................................................aaagucuucacuaUuuucaguu....................   1      1   seq
......................................................................aaagucuucacuacuuucaguA....................   9      1   seq
......................................................................aaagucuucacuacuuucagGu....................   1      1   seq
......................................................................aaagucuucacuacuuucaUuu....................   2      1   seq
......................................................................aaagucuucacuacCuucaguu....................   1      1   seq
......................................................................aaagucuucacuacuuucaguC....................   1      1   seq
......................................................................aGagucuucacuacuuucaguu....................   2      1   seq
......................................................................aaagucuucacuUcuuucaguu....................   1      1   seq
......................................................................Naagucuucacuacuuucaguu....................   2      1   seq
......................................................................aaGgucuucacuacuuucaguu....................   2      1   seq
......................................................................aaagucuucacCacuuucaguu....................   4      1   seq
......................................................................aaagucuuAacuacuuucaguu....................   5      1   seq
......................................................................aaagAcuucacuacuuucaguu....................   7      1   seq
......................................................................aaagucuucacuacuuucaguG....................   3      1   seq
......................................................................aaagucuucacGacuuucaguu....................   1      1   seq
......................................................................aaagucuucacuacuCucaguu....................   5      1   seq
......................................................................aaagucuucacuacuuucaguuG...................   1      1   seq
......................................................................aaagucuucacuacuuucaguuU...................   146    1   seq
......................................................................aaagucuucacuacuuucaguuA...................   100    1   seq
.......................................................................aagucuucacuacuuucaguu....................   3      0   seq
........................................................................agucuucacuacuuucaguu....................   1      0   seq
............................................................................................cggagaugugucuuaauacu   3      0   seq
............................................................................................cggagaugugucuuaauacA   12     1   seq
```
